# tRF‐30‐FP18LPMBQ4NK in Systemic Juvenile Idiopathic Arthritis: A Promising Diagnostic and Disease Activity Biomarker

**DOI:** 10.1111/nyas.70148

**Published:** 2025-12-09

**Authors:** Jiqian Huang, Yuting Pan, Jing Jin, Xiaoyan Shao, Wenjie Zheng, Zhidan Fan, Haiguo Yu

**Affiliations:** ^1^ Department of Rheumatology and Immunology Children's Hospital of Nanjing Medical University Nanjing China; ^2^ Department of Pediatric Rheumatology The Second Affiliated Hospital and Yuying Children's Hospital of Wenzhou Medical University Wenzhou China

**Keywords:** biomarker, disease activity, juvenile idiopathic arthritis, small‐RNA sequencing, transfer RNA fragment

## Abstract

Diagnosing systemic juvenile idiopathic arthritis (sJIA) poses significant challenges. Accumulating evidence has indicated that tRNA‐derived fragments (tRFs) play integral roles in the pathogenesis of numerous diseases. Plasma samples were collected from individuals diagnosed with sJIA and healthy controls (HCs) from two medical centers and divided into training and validation cohorts. Small‐RNA high‐throughput sequencing was employed to investigate the expression profiles of tRFs in the plasma of patients. Aberrantly expressed tRFs in sJIA were validated using quantitative reverse‐transcription PCR (qRT‐PCR). A total of 245 tRFs were differentially expressed in sJIA samples than in HC samples. Through qRT‐PCR validation, tRF‐30‐FP18LPMBQ4NK was identified as a potential biomarker. In the training cohort, plasma levels of tRF‐30‐FP18LPMBQ4NK were significantly higher in patients with sJIA than in HCs. Furthermore, the tRF‐30‐FP18LPMBQ4NK levels in patients in the active disease group were substantially higher than those in the inactive disease group. Additionally, the positive and negative predictive values of the selected tRF in the validation cohort reached 100% and 85%, respectively. Our results suggest that tRF‐30‐FP18LPMBQ4NK can be used as a promising biomarker candidate for sJIA and has the potential to aid in determining disease activity among patients with sJIA.

## 1 Introduction

Juvenile idiopathic arthritis (JIA) is the leading cause of chronic rheumatic disease in children and one of the most disabling conditions in pediatric rheumatology, frequently persisting into adulthood. Manifestations include joint swelling and pain, abnormal growth and development, osteoporosis, psychological disorders, joint deformities, and notable functional disabilities [1, 2]. Epidemiological surveys across Europe and the United States estimate the incidence rate of JIA to be approximately 0.15%. Long‐term monitoring has revealed that only 32.8% of patients achieve clinical remission after 10 years, whereas up to 39% of children experience severe joint disabilities. These outcomes present significant challenges for affected individuals, their families, and society [3]. Systemic juvenile idiopathic arthritis (sJIA) is the most distinct subtype within the seven categories delineated by the International League of Rheumatology Societies (ILAR) [4]. The clinical spectrum includes fever, rashes, splenomegaly, and lymph node enlargement. sJIA resembles an autoinflammatory disease, characterized by significant innate immune aberrations and the absence of autoantibodies. Managing systemic symptoms with pharmacotherapy is challenging, and the disease often progresses to severe arthritis in later stages, leading to childhood disability. In some cases, children experience severe acute‐phase arthritis complicated by the potentially life‐threatening macrophage activation syndrome [5].

In recent years, small RNA fragments known as transfer RNA fragments (tRFs), originating from transfer RNAs (tRNAs), have garnered increasing attention as novel areas of research [6, 7]. tRFs, distinguished by their evolutionary conservation compared to microRNAs (miRNAs) and long noncoding RNAs, are generated through the specific cleavage of tRNA molecules by nucleases such as angiogenin. These small noncoding RNA fragments are involved in controlling key cellular functions such as proliferation, programmed cell death, differentiation, and the inflammatory response [8, 9, 10]. Additionally, tRFs modulate the DNA damage response, influence the stability of mRNAs, and regulate immune cell proliferation [11, 12, 13]. Multiple studies have demonstrated a link between aberrant tRF expression and the outcomes of various conditions, including tumors, metabolic diseases, immune disorders, and inflammatory diseases [14, 15, 16, 17].

However, the involvement of tRFs in sJIA remains incompletely understood. In this study, we investigated the expression patterns of tRFs in the plasma of patients with sJIA and assessed their potential as indicators of disease activity, as well as supplementary diagnostic markers for sJIA.

## 2 Methods

### 2.1 Ethics Statement

The principles outlined in the Declaration of Helsinki were adhered to during the course of this research (1964). All analyses were approved by the Medical Ethics Committee of the Children's Hospital of Nanjing Medical University (202405009‐1) and the Yuying Children's Hospital of Wenzhou Medical University (2024‐K‐376‐01). The requirement for informed consent was waived by both ethics committees, as the study involved only the retrospective analysis of fully deidentified leftover clinical samples and posed no additional risk to the participants.

### 2.2 Patients and Samples

Plasma samples were collected from a cohort comprising 109 children diagnosed with sJIA and 72 healthy controls (HCs) with no documented history of chronic or underlying medical conditions. Samples were sourced from pediatric patients admitted to the Yuying Children's Hospital of Wenzhou Medical University and the Children's Hospital of Nanjing Medical University in China. The first reported patient was recruited in September 2023, and the last reported patient was recruited in July 2024. Anticoagulant‐treated tubes were used to preserve plasma integrity, and diagnosis of sJIA was established according to the criteria set by the ILAR [18].

The JIA cohort from Nanjing was designated as the training group, and patients were further classified based on disease status into two distinct subgroups: those in the active phase (Group A) and those in remission or inactive phase (Group B). In parallel, the cohort from Wenzhou served as the validation group and was similarly stratified into active (Group C) and inactive (Group D) categories. Here, the “active” state refers to JIA patients experiencing current disease activity, whether prior to the initiation of any therapeutic intervention or during a relapse occurring after an initial response to treatment. In contrast, the “inactive” state was defined as a period of inactivity lasting more than 6 months, according to the Wallace criteria [19]. The control group comprised healthy children who underwent physical examinations at the Child Health Clinic. Data on the demographic and clinical characteristics, as well as laboratory findings, were collected and analyzed. These included information on age, sex, arthritis, fever, evanescent rash, lymphadenopathy, hepatomegaly, splenomegaly, serositis, erythrocyte sedimentation rate (ESR), C‐reactive protein (CRP), white blood cell count, hemoglobin, platelets, serum levels of immunoglobulins (IgG, IgM, and IgA), complement (C3, C4), rheumatoid factor, anti‐citrullinated peptide antibodies, and the systemic‐onset Juvenile Arthritis Disease Activity Score 27‐joint reduced count (sJADAS27). Clinical data and blood samples were obtained from all participants. The blood samples were immediately centrifuged at 1000 × *g* for 5 min to separate the plasma, which was then carefully transferred into RNase‐free 1.5 mL polypropylene tubes and stored at −80°C until RNA isolation, which was performed within 1 month.

### 2.3 Small‐RNA Library Preparation and Sequencing

Small‐RNA sequencing was performed on plasma samples obtained from three sJIA patients at diagnosis before initiating any treatment and five HCs. The quality and concentration of each RNA sample were evaluated using agarose gel electrophoresis and quantified with a NanoDrop spectrophotometer (Thermo Fisher Scientific, USA). To mitigate potential interference from RNA modifications in tRFs during small‐RNA sequencing library preparation, total RNA underwent several pretreatments. These included diacylation of the 3′‐aminoacyl (charged) group to generate a 3′‐OH for 3′‐adaptor ligation, conversion of the 3′‐cP (2′,3′‐cyclic phosphate) to a 3′‐OH for 3′‐adaptor ligation, phosphorylation of the 5′‐OH to a 5′‐P for 5′‐adaptor ligation, and demethylation of m1A and m3C to improve reverse‐transcription efficiency (NEBNext Multiplex Small RNA Library Prep Set for Illumina). Library quality and concentration were evaluated using an Agilent BioAnalyzer 2100 (Agilent Technologies, USA), followed by 50‐bp single‐read sequencing on a NextSeq platform (Illumina, USA). Cytosolic tRNA sequences were obtained from the Genomic tRNA Database [20], while mitochondrial tRNA sequences were predicted using tRNAscan‐SE software [21, 22]. To construct mature tRNA libraries, predicted intronic sequences (if present) were removed and a 3′‐terminal “CCA” was appended to each tRNA. For precursor tRNA libraries, 40 nucleotides of genomic sequence flanking each side of the original tRNA sequence were included [23].

### 2.4 RNA Extraction and Quantitative Reverse‐Transcription PCR

To control for variation in plasma RNA extraction and downstream processing, we added a synthetic nonhuman microRNA spike‐in, cel‐miR‐39‐3p (RiboBio, China), to each sample immediately after plasma separation and before adding TRIzol. Routinely, 1 pmol of cel‐miR‐39‐3p (prepared as 200 nM working solution; 5 µL per sample) was pipetted directly into 300 µL of plasma in RNase‐free tubes, vortexed briefly (2–3 s), and kept on ice for ≤5 min prior to TRIzol addition.

Total RNA was extracted from plasma using TRIzol reagent (Vazyme, China). RNA purity and concentration were measured using a NanoDrop 1000 spectrophotometer (Thermo Fisher Scientific), confirming OD 260/280 values within the acceptable range of 1.8–2.0. RNA was then reverse‐transcribed to cDNA using Bulge‐loop quantitative reverse‐transcription PCR (qRT‐PCR) Primer Sets (RiboBio) according to the manufacturer's instructions. Forward primers, 20–40 nucleotides long, contained extensions mimicking small RNA sequences, while universal reverse primers were 16 nucleotides in length. Primer specificity was validated using standard RNAs identical to the target sequences used as positive controls. qRT‐PCR was performed in triplicate on an ABI QuantStudio 3 device (Thermo Fisher Cloud, USA). Expression levels were normalized to cel‐miR‐39‐3p Standard RNA, and relative expression was calculated via the 2^−∆∆Ct^ method. Standard RNA and reverse‐transcription primers of commercial kits (RiboBio) were used. The primer sequences for the target genes were as follows: reverse 5′‐GUGCAGGGUCCGAGGU‐3′; tRF‐30‐FP18LPMBQ4NK, forward 5′‐CCCAGCACGCACCUCGGACCCUGCAC‐3′; tRF‐30‐M2OSRNLNKSEK, forward 5′‐ CCCGUCGGGGACCUCGGACCCUGCAC‐3′; tRF‐23‐897PVP94Z, forward 5′‐ACUCACCACCUCGGACCCUGCAC‐3′; tRF‐28‐79MP9P9NH50E, forward 5′‐AACGUGAUAACCUCGGACCCUGCAC‐3′.

Aliquots of extracted RNA (*n* = 10 representative samples) were treated with RNase‐free DNase I (Servicebio, China) according to the manufacturer's instructions, followed by column cleanup. Reverse transcription reactions were performed in parallel with (+RT) and without reverse transcriptase (−RT) to assess genomic DNA contamination. qPCR was performed as described above; melt curve analysis was used to confirm product specificity.

### 2.5 Statistical Analysis

Sequencing data were processed using the Solexa pipeline v1.8 (Off‐Line Base Caller software, v1.8) for image analysis and base‐calling. Sequence quality was assessed using FastQC, and reads were trimmed to remove 5′‐ and 3′‐adaptor sequences using Cutadapt, following the Illumina quality filter. Trimmed reads were first aligned to mature tRNA sequences, allowing one mismatch. Reads that did not align were subsequently mapped to precursor tRNA sequences using Bowtie, also with a tolerance of a single mismatch. Any remaining reads were mapped to reference miRNA sequences using miRDeep2, also with one‐mismatch tolerance. Expression levels of tRFs and miRNAs were quantified based on read counts, and differential expression analysis was conducted using the EdgeR package [24]. Statistical visualizations—including principal component analysis, pie charts, Venn diagrams, hierarchical clustering, and scatter plots—were generated using R software for statistical computing.

Nonsequencing data were analyzed using IBM SPSS Statistics 22.0 and GraphPad Prism version 9. Data are presented as means ± standard deviation from at least three independent experiments, unless otherwise stated. For qRT‐PCR analyses, comparisons between two groups were made using two‐tailed unpaired Student's *t*‐tests. Spearman's correlation test was employed for correlation analysis. All statistical tests were two‐sided, and statistical significance is reported at *p* < 0.05. The following significance thresholds were used: *p < 0.05, **p < 0.01, and ****p < 0.0001.

## 3 Results

### 3.1 Participant Characteristics

All participants were of Han ethnicity, with no significant differences in age or sex between patients with sJIA and HCs, as shown in Table S1. The clinical features of the sJIA patients across the four subgroups are presented in Table S2. Compared with groups B and D, those in the active state of sJIA exhibited no differences in age or sex.

### 3.2 Differentially Expressed tRFs in Plasma of Patients With sJIA and HCs

**FIGURE 1:**
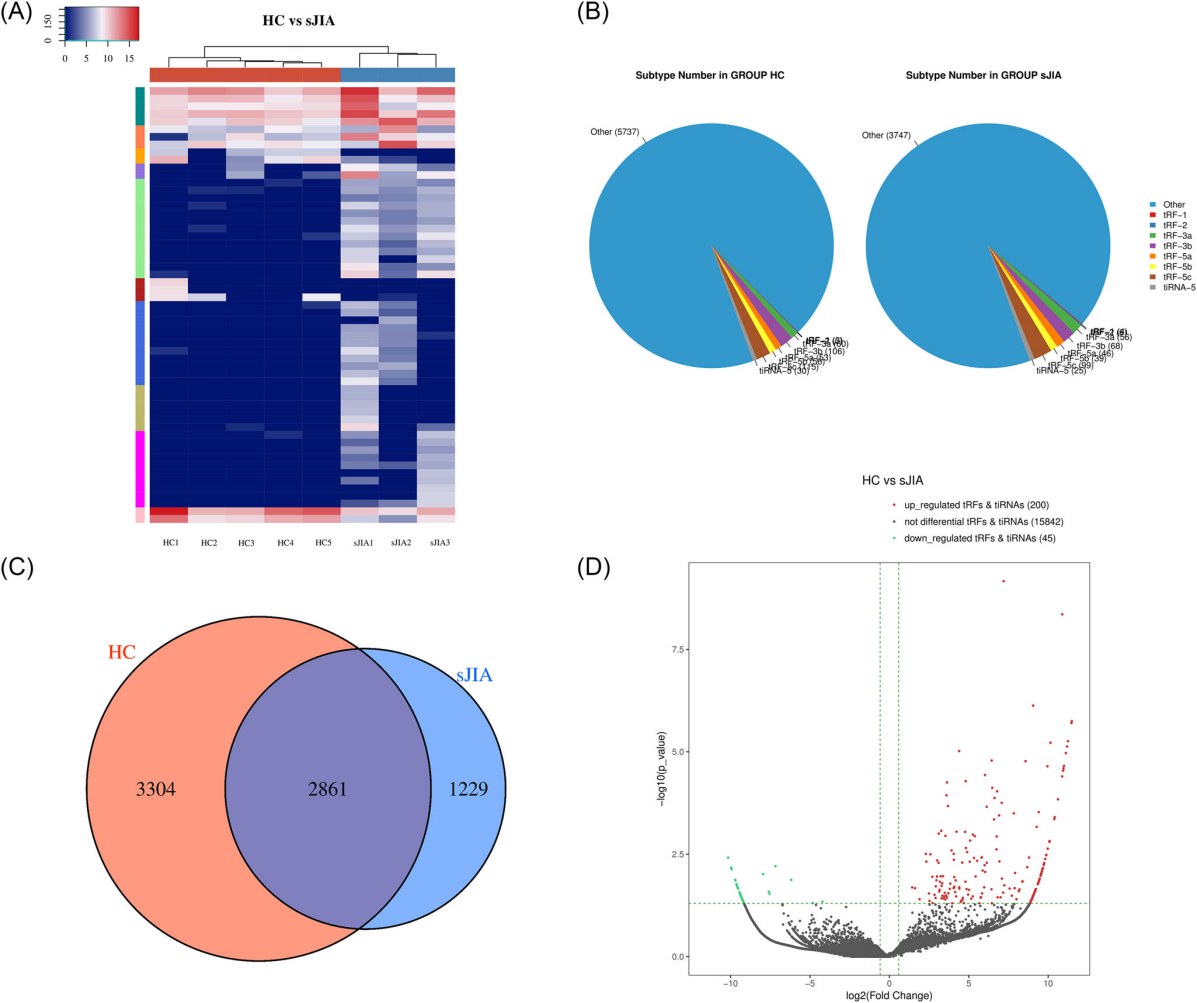
Expression profiles of tRFs in the plasma of sJIA and HCs. (A) Heat map depicting the differentially expressed tRFs between five HC and three sJIA plasma samples. The color scale on the upper left indicates relative expression levels, with red representing upregulation and blue indicating downregulation. (B) Pie charts illustrating the distribution of tRF subtypes, where the CPM for individual samples or the group average did not fall below 20. Separate distributions are shown for the HC (left) and sJIA (right) groups. (C) Venn diagram showing the overlap and specificity of tRF expression between the HC and sJIA groups. Commonly expressed tRFs have CPM values exceeding 20 in both groups, while specifically expressed tRFs have CPM values >20 in one group and <20 in the other. (D) Volcano plot of differentially expressed tRFs between HC and sJIA samples. The *x*‐axis represents log_2_ fold changes, while the *y*‐axis represents −log_10_ transformed *p*‐values. Red and green dots indicate statistically significant upregulated and downregulated tRFs, respectively, with a fold change ≥1.5 and *p*‐value ≤0.05. Gray dots represent tRFs that did not meet the threshold for differential expression.

Figure S1 shows a schematic of the workflow. To assess the differential expression of tRFs in the plasma of sJIA patients, small‐RNA sequencing was conducted on plasma samples from three sJIA patients and five HCs, followed by clustering analysis (Figure 1A). Our sequencing data revealed 245 tRFs with significant differential expression (fold change ≥ 2 or ≤ 0.5 and *p* < 0.05), comprising 200 upregulated and 45 downregulated tRFs. These tRFs were classified into subtypes according to their length and size. A pie chart illustrates the distribution of each tRF subtype in patients with sJIA and HCs, revealing a notable increase in tRF‐3a expression in the sJIA group (Figure 1B). A Venn diagram shows the most commonly and uniquely expressed tRFs (Figure 1C), while a volcano plot visualizes tRFs with significant and large‐magnitude changes between the two sample groups (Figure 1D).

### 3.3 qRT‐PCR Validation of sJIA‐Related tRF Dysregulation

**FIGURE 2:**
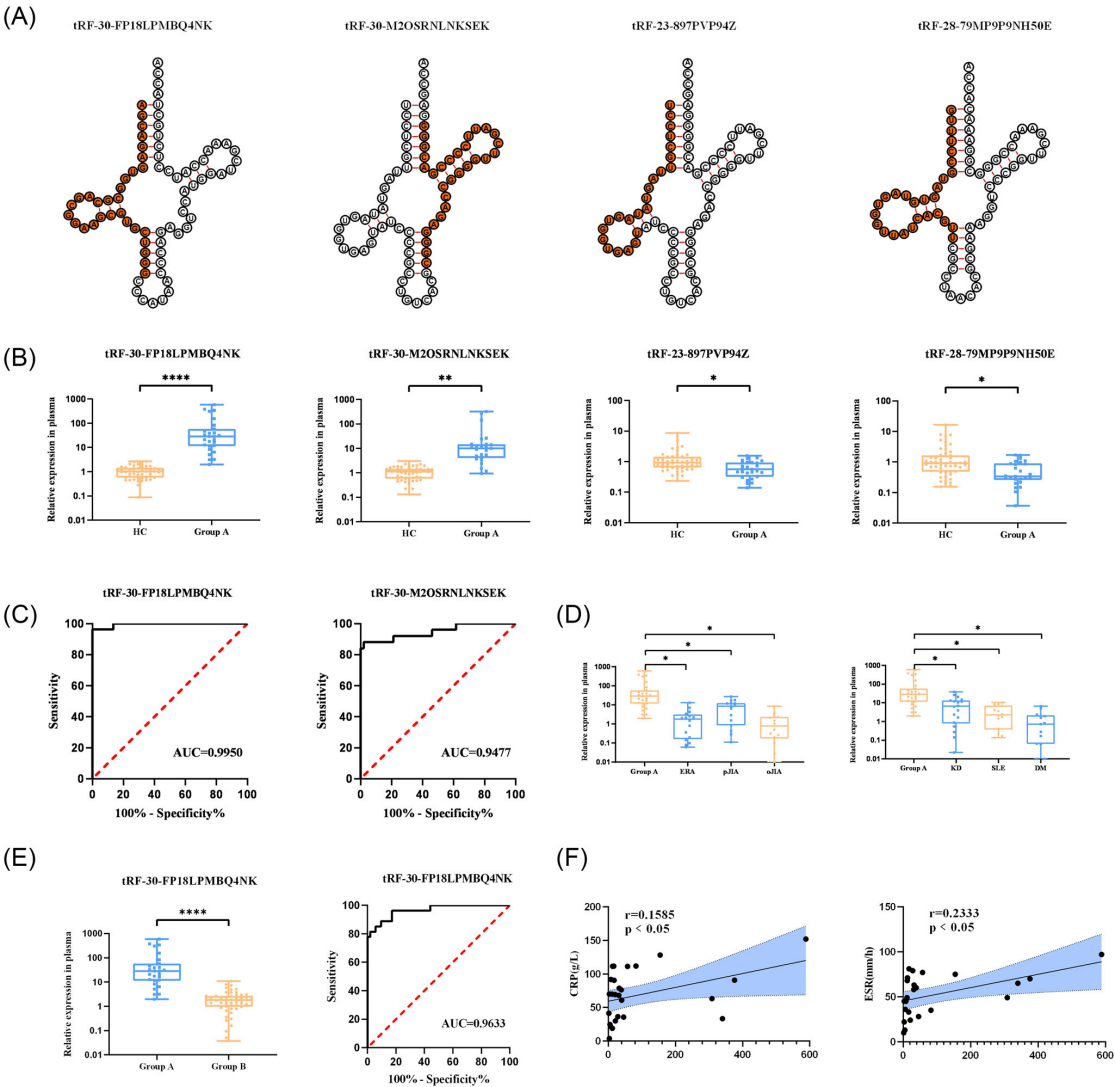
Diagnostic and prognostic value of the identified tRFs in the training cohort. (A) Schematic representation of the location of tRFs in the cloverleaf secondary structure of tRNAs. (B) Quantitative expression levels of tRFs in the plasma of sJIA patients and HCs, as measured by qRT‐PCR. (C) ROC curves for tRF‐30‐FP18LPMBQ4NK and tRF‐30‐M2OSRNLNKSEK. (D) Expression levels of tRF‐30‐FP18LPMBQ4NK in the plasma of patients with other subtypes of JIA and other rheumatic diseases. (E) Comparison of tRF‐30‐FP18LPMBQ4NK expression levels in plasma between patients in the active and inactive states of sJIA, and the ROC curve for it. (F) Correlation analysis between plasma tRF‐30‐FP18LPMBQ4NK levels and CRP and ESR.

Two significantly upregulated tRFs (tRF‐30‐FP18LPMBQ4NK and tRF‐30‐M2OSRNLNKSEK) and two significantly downregulated tRFs (tRF‐23‐897PVP94Z and tRF‐28‐79MP9P9NH50E) were selected from the plasma of sJIA patients. The sequences of the parent tRNAs for these selected tRFs are shown in Table S3. The positions of the selected tRFs are depicted in the cloverleaf secondary structures of their corresponding tRNAs (Figure 2A). To validate the expression of these four tRFs, we performed qRT‐PCR in a larger cohort comprising 79 sJIA patients and 52 HCs (Figure 2B). The expression levels of tRF‐30‐FP18LPMBQ4NK and tRF‐28‐79MP9P9NH50E were significantly higher in sJIA patients compared with HCs (*p* < 0.0001 and *p* < 0.01, respectively), whereas the expression levels of tRF‐23‐897PVP94Z and tRF‐28‐79MP9P9NH50E were significantly lower in sJIA patients (both *p* < 0.05).

To exclude the possibility of genomic DNA contamination, we performed DNase treatment and paired −RT controls on a representative subset of samples (*n* = 10). In all cases, the −RT reactions showed no detectable amplification (Ct undetermined or Ct ≥ 35). Both DNase‐treated and untreated +RT reactions yielded robust amplification, and the ΔCt between +RT and −RT reactions were ≥14.845, supporting the absence of detectable DNA‐derived signal. Ct values were comparable between DNase‐treated and untreated +RT groups, indicating that DNase treatment did not affect RNA detection. The specific Ct values for all samples are presented in Table S4, and representative amplification and melt curves are shown in Figures S2 and S3.

### 3.4 Diagnostic and Disease Activity Values of Identified tRFs

The two upregulated tRFs exhibited higher relative expression levels than the downregulated tRFs, which prompted us to select the upregulated tRFs for further analysis. Diagnostic performance was assessed using receiver operating characteristic (ROC) curves. The area under the curve (AUC) for tRF‐30‐FP18LPMBQ4NK was 0.9950 (95% CI, 0.9843–1.0000; *p* < 0.0001) (Figure 2C), whereas that for the tRF‐30‐M2OSRNLNKSEK panel was 0.9477 (95% CI, 0.8865–1.0000; *p* < 0.0001) (Figure 2C). Additionally, we found that the elevation of tRF‐30‐FP18LPMBQ4NK remained specific compared with other subtypes of JIA and other rheumatic diseases (Figure 2D). These findings suggest that tRF‐30‐FP18LPMBQ4NK has superior diagnostic specificity and accuracy.

qRT‐PCR was also performed to confirm the levels of tRF‐30‐FP18LPMBQ4NK in the plasma of children with various disease activities (Figure 2E). The results revealed significantly higher levels in Group A than in Group B, indicating that tRF‐30‐FP18LPMBQ4NK may function as a marker for assessing disease activity in sJIA. ROC curve analysis yielded an AUC of 0.9633. Further integration with clinical data revealed a positive correlation between tRF‐30‐FP18LPMBQ4NK expression in patients with active disease and CRP and ESR levels (Figure 2F). Therefore, the abundance of tRF‐30‐FP18LPMBQ4NK was strongly associated with key clinical markers, such as ESR and CRP. These findings suggest that increased levels of tRF‐30‐FP18LPMBQ4NK may be crucial in the inflammatory pathogenesis of sJIA.

### 3.5 Biomarker Validation

**FIGURE 3:**
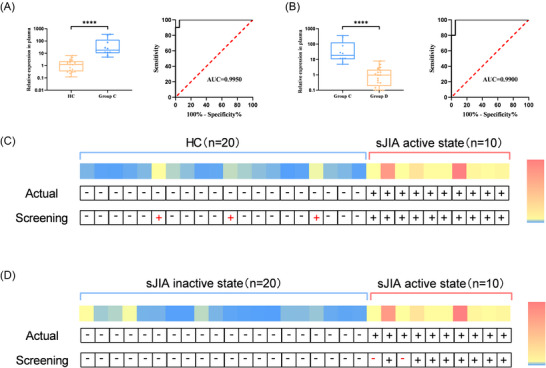
Validation of differential expression levels of tRF‐30‐FP18LPMBQ4NK in sJIA. (A) Quantitative expression levels of tRFs in the plasma of sJIA patients and HCs, and the ROC curve for it. (B) Comparison of tRF‐30‐FP18LPMBQ4NK expression levels in plasma between patients in the active and inactive states of sJIA, and the ROC curve for it. (C) Diagnostic potency of tRF‐30‐FP18LPMBQ4NK for detection of sJIA patients in a screening test with a cutoff concentration at 2.873. (D) Efficacy of tRF‐30‐FP18LPMBQ4NK in assessing disease activity in sJIA patients, with a cutoff concentration at 8.154 in the screening test.

**FIGURE 4:**
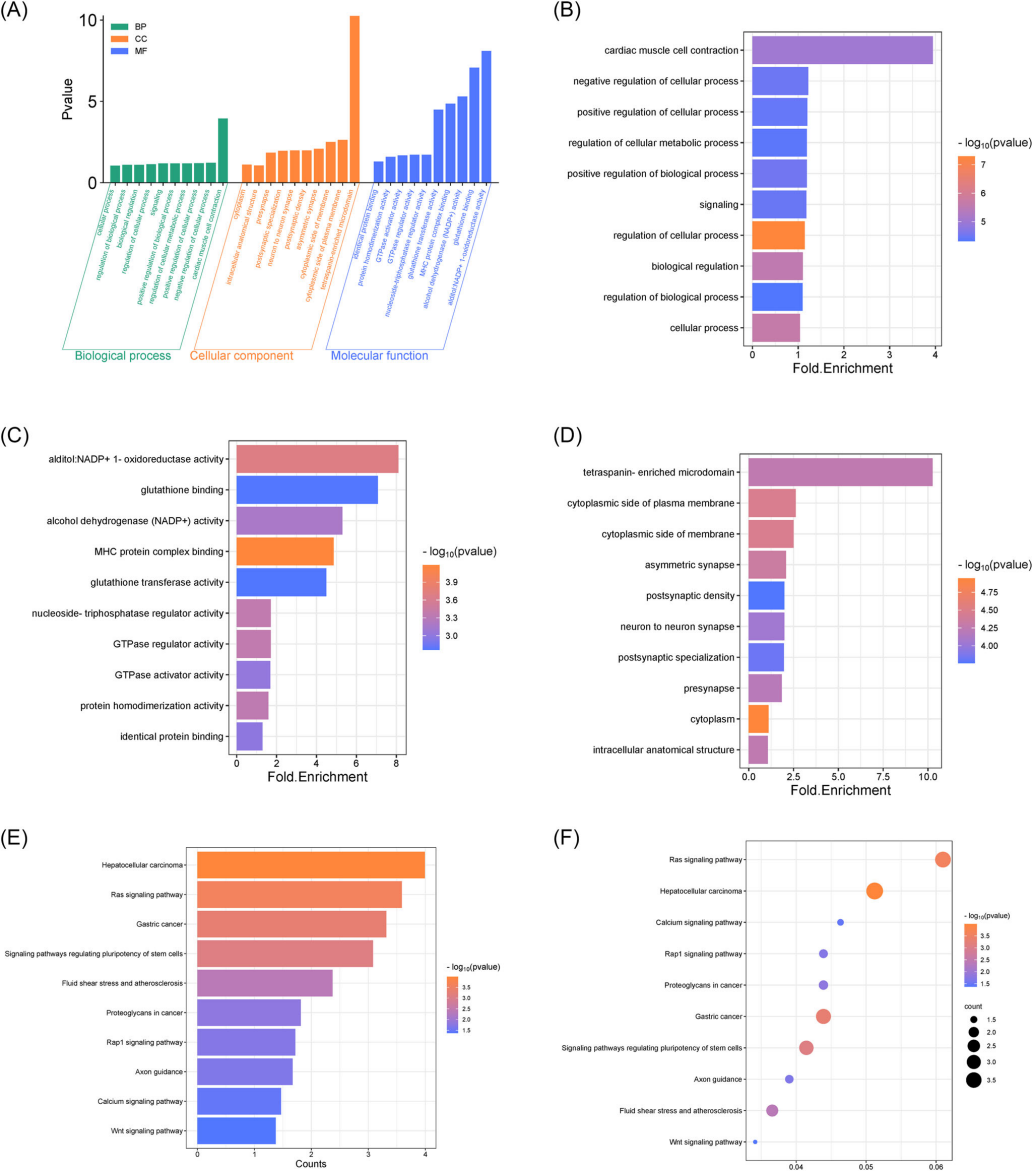
Bioinformatics analysis of identified tRF‐30‐FP18LPMBQ4NK. (A) GO enrichment analysis of tRF‐30‐FP18LPMBQ4NK based on three aspects: (B) biological processes, (C) molecular functions, (D) cellular components. (E, F): KEGG pathway analysis of tRF‐30‐FP18LPMBQ4NK. Abbreviations: GO, Gene Ontology; KEGG, Kyoto Encyclopedia of Genes and Genomes.

To rapidly screen patients with sJIA, we set the cutoff value for tRF‐30‐FP18LPMBQ4NK expression at 2.873, and for assessing disease activity, the threshold was 8.154, both of which were identified as the optimal thresholds from the ROC curve. As shown in Figure 3A,B, the increasing trend of tRF‐30‐FP18LPMBQ4NK in the validation cohort closely mirrored that in the training cohort. The ROC curve further demonstrated the strong diagnostic performance of this method. tRF30‐FP18LPMBQ4NK demonstrated high diagnostic and predictive values for sJIA, with an accuracy of 90% in a sample set of 30 patients, emphasizing its potential for accurate clinical diagnosis (Figure 3C). In terms of assessing disease activity, the accuracy reached 93%, reinforcing its usefulness in monitoring disease activity in children with sJIA (Figure 3D). As illustrated in Figure 4, tRF‐30‐FP18LPMBQ4NK achieved an accuracy of 90% in predicting sJIA based on 30 samples and 93% in distinguishing the active state of sJIA from the inactive state. These findings suggest that tRF‐30‐FP18LPMBQ4NK has great potential for the precise diagnosis of sJIA, supporting its future application in clinical practice. Furthermore, we compared our qRT‐PCR‐based detection of tRF‐30‐FP18LPMBQ4NK to existing biomarkers for sJIA diagnosis. The comparison, summarized in Table 1, includes AUC, sensitivity, specificity, noninvasiveness, and clinical screening capability. The results showed that our method achieved the highest sensitivity and specificity among all tested approaches. Furthermore, using the selected cutoff value, we achieved a screening accuracy of up to 90% for sJIA, surpassing the accuracy of all current methods.

**TABLE 1:** Comparing the present method to the existing methods for sJlA diagnosis.

Method	Marker	AUC	Sensitivity	Specificity	Sample	Clinical screening	Ref.
qRT‐PCR	tRF‐30‐FP18LPMBQ4NK	0.995	96.3%	98.1%	Plasma	PPV=100%.NPV=85%	Our method
ELISA	MRP8/14	0.874	74.6%	88.2%	Serum	PPV=73.3%.NPV=88.9%	[25]
Machine learning	ALDH1A1.CEACAM1 YBX3.SLC6A8	0.990	86.4%	94.1%	Whole blood	/	[26]
Flow cytometry	PD‐1	0.720−0.880	/	/	Whole blood	/	[27]
qRT‐PCR	miR‐26a	0.878	/	/	Plasma	/	[28]
ELISA	IL‐10	0.862	91.2%	66.7%	Serum	/	[29]
ELISA	S100A8/9	0.900	74.0%	91.0%	Serum	/	[30]
ELISA	S100A12	0.900	71.0%	89.0%	Serum	/	[30]

Abbreviations: AUC, area under the curve; NPV, negative predictive value; PPV, positive predictive value.

### 3.6 Bioinformatics Analysis of tRF‐30‐FP18LPMBQ4NK

The fragment tRF‐30‐FP18LPMBQ4NK, derived from mature tRNA, is a tRF‐5c fragment. Gene Ontology (GO) and Kyoto Encyclopedia of Genes and Genomes (KEGG) pathway analyses were performed to further investigate its function. The GO analysis (Figure 4A) comprised three terms: biological processes (Figure 4B), molecular functions (Figure 4C), and cellular components (Figure 4D). Our analyses revealed that the target genes of tRF30‐FP18LPMBQ4NK play a broad role in regulating cellular activities, biological processes, and cell signaling pathways (Figure 4B). The KEGG pathway enrichment analysis further indicated that these genes were mainly associated with Ras, calcium, Rap1, and other cancer‐related signaling pathways (Figure 4E,F).

## 4 Discussion

Diagnosing sJIA remains a considerable clinical challenge, as there are currently no reliable noninvasive biomarkers available. tRFs are small noncoding RNA molecules, exhibiting high conservation across species, which emphasizes their likely biological importance [31]. Recent studies have uncovered previously unrecognized roles of tRFs in the regulation of key physiological and pathological processes, including neurodegeneration, cancer, viral infections, and rheumatic diseases [14, 15, 16, 17]. Owing to their varied functional roles, tRFs present as promising candidates for diagnostic markers and potential treatment targets in a range of diseases.

This study employed small‐RNA high‐throughput sequencing to examine the expression patterns of tRFs in the plasma of sJIA patients. A total of 200 tRFs were found to be upregulated, whereas 45 were downregulated compared with those in HCs, as identified through RNA sequencing. qRT‐PCR validation confirmed the upregulation and downregulation of two tRFs each in patients with sJIA. In the training cohort, tRF‐30‐FP18LPMBQ4NK demonstrated strong diagnostic potential, with an AUC of 0.9950, sensitivity of 96.3%, and specificity of 98.1% in differentiating patients with sJIA from HCs. In the validation cohort, a threshold of 2.873, identified as the optimal cutoff from the ROC curve, was used for sJIA detection. As illustrated in Figure 4, tRF‐30‐FP18LPMBQ4NK achieved an accuracy of 90% in predicting sJIA based on 30 samples and achieved an accuracy of 93% in distinguishing the active state of sJIA from the inactive state. These findings suggest that tRF‐30‐FP18LPMBQ4NK has great potential for the precise diagnosis of sJIA, supporting its future application in clinical practice. Additionally, the abundance of tRF‐30‐FP18LPMBQ4NK was strongly associated with ESR, CRP, and other key clinical markers of sJIA. These findings, therefore, strongly indicate the biomarker potential of tRF‐30‐FP18LPMBQ4NK as its increased levels coincide with the inflammatory pathogenesis of sJIA.

The Ras signaling pathway is a crucial regulator of cell proliferation, survival, and differentiation [32]. When activated by growth factors, cytokines, or other extracellular signals, Ras triggers a cascade involving the RAF/MEK/ERK pathway, resulting in alterations in the expression of genes that control cell cycle progression and inflammatory responses [33, 34]. This pathway also interacts with other key signaling pathways, such as phosphoinositide 3‐kinase/protein kinase B and nuclear factor kappa B (NF‐κB), playing major roles in both normal cellular functions and pathological conditions, such as cancer and inflammation [35, 36]. Dysregulation of Ras signaling can lead to uncontrolled cell growth and heightened inflammatory responses, which play a role in various diseases, including autoimmune disorders [37]. The Ras signaling pathway could play a crucial role in the inflammatory environment of sJIA by enhancing cytokine production, supporting neutrophil activation, and influencing NF‐κB‐mediated inflammasome priming. Additional analysis of the downstream regulatory mechanisms of tRF‐30‐FP18LPMBQ4NK highlighted its role in the Ras signaling pathway. Our findings indicate that tRF‐30‐FP18LPMBQ4NK, through its regulation of Ras target genes, may act as a significant molecular regulator that contributes to the disease process by modulating these key pathways. Emerging evidence supports that 5′ tRFs (including tRNA halves) are produced under stress or inflammatory conditions, especially by activated immune cells [38, 39]. Given the known hyperactivation of innate immune cells in sJIA, and the significant correlation of tRF‐30‐FP18LPMBQ4NK with inflammatory markers (CRP and ESR) in our cohort, it is plausible that tRF‐30‐FP18LPMBQ4NK could be produced and released by activated monocytes or neutrophils in sJIA, potentially via EVs or RNA–protein complexes. Further investigation into the role of tRF‐30‐FP18LPMBQ4NK in Ras signaling and its cellular origin could provide new insights into the pathogenesis of sJIA and reveal potential therapeutic targets.

A limitation of this study is that the original experiments did not include explicit −RT controls. To address this, we performed DNase treatment and paired −RT controls on representative samples, which confirmed that genomic DNA contamination did not significantly affect the measured tRF signals. In future studies, we will incorporate DNase treatment and −RT controls as a standard procedure to further ensure the specificity of qPCR measurements.

## 5 Conclusion

In summary, our study investigated the unique tRFs in patients with sJIA and HCs via the integration of small‐RNA sequencing and qRT‐PCR for the fast detection and screening of sJIA. We demonstrated that tRF‐30‐FP18LPMBQ4NK is a promising diagnostic and disease activity biomarker.

## Author Contributions

Haiguo Yu, Zhidan Fan, and Wenjie Zheng conceptualized and designed the study, acquired funding, provided resources, administered and supervised the project, and finally reviewed and edited the manuscript. Jiqian Huang and Yuting Pan handled software and visualization tasks. Jing Jin and Xiaoyan Shao conducted validation and contributed to the methodology. All authors have read and approved the final version of the manuscript.

## Funding

This study was supported by the National Key R&D Program of China (grant number: 2021YFC2702000), National Natural Science Foundation of China (grant numbers: 82271838 and 81771762), and Jiangsu Provincial Health Commission (grant numbers: M2021080 and M2022018), Zhejiang Provincial Natural Science Foundation of China (grant number: LY23H100002).

## Conflicts of Interest

The authors declare that they have no conflicts of interest related to this work. All authors have completed the International Committee of Medical Journal Editors (ICMJE) uniform disclosure form, and no potential conflicts of interest were reported.

## Ethical Approval Statement

All analyses were approved by the Medical Ethics Committee of the Children's Hospital of Nanjing Medical University (202405009‐1) and the Yuying Children's Hospital of Wenzhou Medical University (2024‐K‐376‐01). All procedures involving human participants were conducted in accordance with the Declaration of Helsinki. Written informed consent was obtained from the parents or legal guardians of all pediatric participants, and assent was obtained from the children when appropriate. This manuscript does not contain any individual person's identifiable data; therefore, consent for publication was not required.

## Supporting information



Supplementary Materials: nyas70148‐sup‐0001‐SuppMat.docx

## Data Availability

The raw data that support the findings of this study are available from the corresponding author upon reasonable request.

## References

[1] C. Hinze , F. Gohar , and D. Foell , “Management of Juvenile Idiopathic Arthritis: Hitting the Target,” Nature Reviews Rheumatology 11 (2015): 290–300. 25561365 10.1038/nrrheum.2014.212

[2] A. Ravelli and A. Martini , “Juvenile Idiopathic Arthritis,” Lancet 369 (2007): 767–778. 17336654 10.1016/S0140-6736(07)60363-8

[3] F. Fantini , V. Gerloni , M. Gattinara , et al., “Remission in Juvenile Chronic Arthritis: A Cohort Study of 683 Consecutive Cases With a Mean 10 Year Followup,” Journal of Rheumatology 30 (2003): 579–584. 12610820

[4] R. E. Petty , T. R. Southwood , J. Baum , et al., “Revision of the Proposed Classification Criteria for Juvenile Idiopathic Arthritis: Durban, 1997,” Journal of Rheumatology 25 (1998): 1991–1994. 9779856

[5] J. J. Y. Lee and R. Schneider , “Systemic Juvenile Idiopathic Arthritis,” Pediatric Clinics of North America 65 (2018): 691–709. 30031494 10.1016/j.pcl.2018.04.005

[6] D. Green , W. D. Fraser , and T. Dalmay , “Transfer RNA‐Derived Small RNAs in the Cancer Transcriptome,” Pflugers Archiv: European Journal of Physiology 468 (2016): 1041–1047. 27095039 10.1007/s00424-016-1822-9 PMC4893054

[7] S. Li , Z. Xu , and J. Sheng , “tRNA‐Derived Small RNA: A Novel Regulatory Small Non‐Coding RNA,” Genes (Basel) 9 (2018): 246. 29748504 10.3390/genes9050246 PMC5977186

[8] S. P. Keam , A. Sobala , S. T. Have , et al., “tRNA‐Derived RNA Fragments Associate With Human Multisynthetase Complex (MSC) and Modulate Ribosomal Protein Translation,” Journal of Proteome Research 16 (2017): 413–420. 27936807 10.1021/acs.jproteome.6b00267

[9] Y. Fu , I. Lee , Y. S. Lee , et al., “Small Non‐Coding Transfer RNA‐Derived RNA Fragments (tRFs): Their Biogenesis, Function and Implication in Human Diseases,” Genomics & Informatics 13 (2015): 94–101. 26865839 10.5808/GI.2015.13.4.94 PMC4742329

[10] P. Kumar , S. B. Mudunuri , J. Anaya , et al., “tRFdb: A Database for Transfer RNA Fragments,” Nucleic Acids Research 43 (2015): D141–145. 25392422 10.1093/nar/gku1138 PMC4383946

[11] S. Lu , X. Wei , L. Tao , et al., “A Novel tRNA‐Derived Fragment tRF‐3022b Modulates Cell Apoptosis and M2 Macrophage Polarization via Binding to Cytokines in Colorectal Cancer,” Journal of Hematology & Oncology 15 (2022): 176. 36527118 10.1186/s13045-022-01388-z PMC9756499

[12] W. Chen , W. Peng , R. Wang , et al., “Exosome‐Derived tRNA Fragments tRF‐GluCTC‐0005 Promotes Pancreatic Cancer Liver Metastasis by Activating Hepatic Stellate Cells,” Cell Death & Disease 15 (2024): 102. 38291031 10.1038/s41419-024-06482-3 PMC10827722

[13] X. Gao , Y. Qiao , S. Li , et al., “tRF‐003634 Alleviates Adriamycin‐Induced Podocyte Injury by Reducing the Stability of TLR4 mRNA,” PLoS ONE 18 (2023): e0293043. 37856510 10.1371/journal.pone.0293043 PMC10586663

[14] P. Wang , Z. Fu , Y. Liu , et al., “tRF‐21‐LNK8KEP1B as a Potential Novel Diagnostic Biomarker for Enthesitis‐Related Arthritis,” International Immunopharmacology 124 (2023): 110820. 37660592 10.1016/j.intimp.2023.110820

[15] A. R. Soares and M. Santos , “Discovery and Function of Transfer RNA‐Derived Fragments and Their Role in Disease,” Wiley Interdisciplinary Reviews‐RNA 8 (2017): e1423. 10.1002/wrna.1423 28608481

[16] Y. Hu , A. Cai , J. Xu , et al., “An Emerging Role of the 5' Termini of Mature tRNAs in Human Diseases: Current Situation and Prospects,” Biochimica et Biophysica Acta ‐ Molecular Basis of Disease 1868 (2022): 166314. 34863896 10.1016/j.bbadis.2021.166314

[17] X. Yu , Y. Xie , S. Zhang , et al., “tRNA‐Derived Fragments: Mechanisms Underlying Their Regulation of Gene Expression and Potential Applications as Therapeutic Targets in Cancers and Virus Infections,” Theranostics 11 (2021): 461–469. 33391486 10.7150/thno.51963 PMC7681095

[18] R. E. Petty , T. R. Southwood , P. Manners , et al., “International League of Associations for Rheumatology Classification of Juvenile Idiopathic Arthritis: Second Revision, Edmonton, 2001,” Journal of Rheumatology 31 (2004): 390–392. 14760812

[19] C. A. Wallace , E. H. Giannini , B. Huang , et al., “American College of Rheumatology Provisional Criteria for Defining Clinical Inactive Disease in Select Categories of Juvenile Idiopathic Arthritis,” Arthritis Care & Research 63 (2011): 929–936. 21717596 10.1002/acr.20497

[20] P. P. Chan and T. M. Lowe , “GtRNAdb 2.0: An Expanded Database of Transfer RNA Genes Identified in Complete and Draft Genomes,” Nucleic Acids Research 44 (2016): D184–189. 26673694 10.1093/nar/gkv1309 PMC4702915

[21] T. M. Lowe and P. P. Chan , “tRNAscan‐SE On‐Line: Integrating Search and Context for Analysis of Transfer RNA Genes,” Nucleic Acids Research 44 (2016): W54–57. 27174935 10.1093/nar/gkw413 PMC4987944

[22] T. M. Lowe and S. R. Eddy , “tRNAscan‐SE: A Program for Improved Detection of Transfer RNA Genes in Genomic Sequence,” Nucleic Acids Research 25 (1997): 955–964. 9023104 10.1093/nar/25.5.955 PMC146525

[23] S. R. Selitsky and P. Sethupathy , “tDRmapper: Challenges and Solutions to Mapping, Naming, and Quantifying tRNA‐Derived RNAs From Human Small RNA‐Sequencing Data,” BMC Bioinformatics [Electronic Resource] 16 (2015): 354. 26530785 10.1186/s12859-015-0800-0 PMC4632369

[24] M. D. Robinson , D. J. McCarthy , and G. K. Smyth , “edgeR: A Bioconductor Package for Differential Expression Analysis of Digital Gene Expression Data,” Bioinformatics 26 (2010): 139–140. 19910308 10.1093/bioinformatics/btp616 PMC2796818

[25] C. Park , M. Miranda‐Garcia , R. Berendes , et al., “MRP8/14 Serum Levels as Diagnostic Markers for Systemic Juvenile Idiopathic Arthritis in Children With Prolonged Fever,” Rheumatology 61 (2022): 3082–3092. 34559193 10.1093/rheumatology/keab729

[26] P. Ding , Y. Du , X. Jiang , et al., “Establishment and Analysis of a Novel Diagnostic Model for Systemic Juvenile Idiopathic Arthritis Based on Machine Learning,” Pediatric Rheumatology 22 (2024): 18. 38243323 10.1186/s12969-023-00949-x PMC10797915

[27] S. Shenoi , J. N. Ou , C. Ni , et al., “Comparison of Biomarkers for Systemic Juvenile Idiopathic Arthritis,” Pediatric Research 78 (2015): 554–559. 26267155 10.1038/pr.2015.144

[28] J. Sun , M. Feng , F. Wu , et al., “Plasma miR‐26a as a Diagnostic Biomarker Regulates Cytokine Expression in Systemic Juvenile Idiopathic Arthritis,” Journal of Rheumatology 43 (2016): 1607–1614. 27252421 10.3899/jrheum.150593

[29] Y. Peng , X. Liu , Z. Duan , et al., “The Association of Serum IL‐10 Levels With the Disease Activity in Systemic‐Onset Juvenile Idiopathic Arthritis Patients,” Mediators of Inflammation 2021 (2021): 6650928. 33824623 10.1155/2021/6650928 PMC8007368

[30] N. Aljaberi , E. Tronconi , G. Schulert , et al., “The Use of S100 Proteins Testing in Juvenile Idiopathic Arthritis and Autoinflammatory Diseases in a Pediatric Clinical Setting: A Retrospective Analysis,” Pediatric Rheumatology 18 (2020): 7. 31948488 10.1186/s12969-020-0398-2 PMC6966841

[31] S. Lalande , R. Merret , T. Salinas‐Giege , et al., “Arabidopsis tRNA‐Derived Fragments as Potential Modulators of Translation,” RNA Biology 17 (2020): 1137–1148. 31994438 10.1080/15476286.2020.1722514 PMC7549631

[32] M. Malumbres and M. Barbacid , “RAS Oncogenes: The First 30 Years,” Nature Reviews Cancer 3 (2003): 459–465. 12778136 10.1038/nrc1097

[33] J. A. McCubrey , L. S. Steelman , W. H. Chappell , et al., “Roles of the Raf/MEK/ERK Pathway in Cell Growth, Malignant Transformation and Drug Resistance,” Biochimica Et Biophysica Acta 1773 (2007): 1263–1284. 17126425 10.1016/j.bbamcr.2006.10.001 PMC2696318

[34] A. S. Dhillon , S. Hagan , O. Rath , et al., “MAP Kinase Signalling Pathways in Cancer,” Oncogene 26 (2007): 3279–3290. 17496922 10.1038/sj.onc.1210421

[35] M. C. Mendoza , E. E. Er , and J. Blenis , “The Ras‐ERK and PI3K‐mTOR Pathways: Cross‐Talk and Compensation,” Trends in Biochemical Sciences 36 (2011): 320–328. 21531565 10.1016/j.tibs.2011.03.006 PMC3112285

[36] G. Courtois and T. D. Gilmore , “Mutations in the NF‐kappaB Signaling Pathway: Implications for Human Disease,” Oncogene 25 (2006): 6831–6843. 17072331 10.1038/sj.onc.1209939

[37] M. Sadeghi Shaker , M. Rokni , M. Mahmoudi , et al., “Ras Family Signaling Pathway in Immunopathogenesis of Inflammatory Rheumatic Diseases,” Frontiers in Immunology 14 (2023): 1151246. 37256120 10.3389/fimmu.2023.1151246 PMC10225558

[38] A. A. Artamonov , K. A. Kondratov , E. A. Bystritsky , et al., “Changes in the Repertoire of tRNA‐Derived Fragments in Different Blood Cell Populations,” Life (Basel) 10 (2024): 1279. 10.3390/life14101294 PMC11509557 39459595

[39] K. Pawar , M. Shigematsu , S. Sharbati , et al., “Infection‐Induced 5'‐Half Molecules of tRNAHisGUG Activate Toll‐Like Receptor 7,” PLoS Biology 18 (2020): e3000982. 33332353 10.1371/journal.pbio.3000982 PMC7745994

