# Supplementary material for: tRF‐30‐FP18LPMBQ4NK in Systemic Juvenile Idiopathic Arthritis: A Promising Diagnostic and Disease Activity Biomarker
Source: Ann N Y Acad Sci. 2025 Dec 9;1556(1):e70148. doi: 10.1111/nyas.70148 (PMC12917932; doi:10.1111/nyas.70148)
Supplement: Supplementary file 1 — Supplementary Materials: nyas70148‐sup‐0001‐SuppMat.docx [file NYAS-1556-0-s001.docx]

**tRF-30-FP18LPMBQ4NK in systemic juvenile idiopathic arthritis: A promising diagnostic and disease activity biomarker**

Jiqian Huang^1,2,a^, Yuting Pan^1,a^, Jing Jin^1^, Xiaoyan Shao^1^, Wenjie Zheng^2^*, Zhidan Fan^1^*, Haiguo Yu^1^*

^1^Department of Rheumatology and Immunology, Children's Hospital of Nanjing Medical University, Nanjing, China

^2^Department of Pediatric Rheumatology, The Second Affiliated Hospital and Yuying Children's Hospital of Wenzhou Medical University, Wenzhou, China

^a^ These authors contributed equally to this work.

* **Correspondence:** Haiguo Yu, Zhidan Fan, and Wenjie Zheng

E-mail: haiguo_yu@njmu.edu.cn (HGY); zhidanfan@njmu.edu.cn (ZDF); [wzwjzheng@sina.com](mailto:wzwjzheng@sina.com) (WJZ)

Tel: 13770757631; 13382022763; 13506662927

Fax: 86+025-83116830


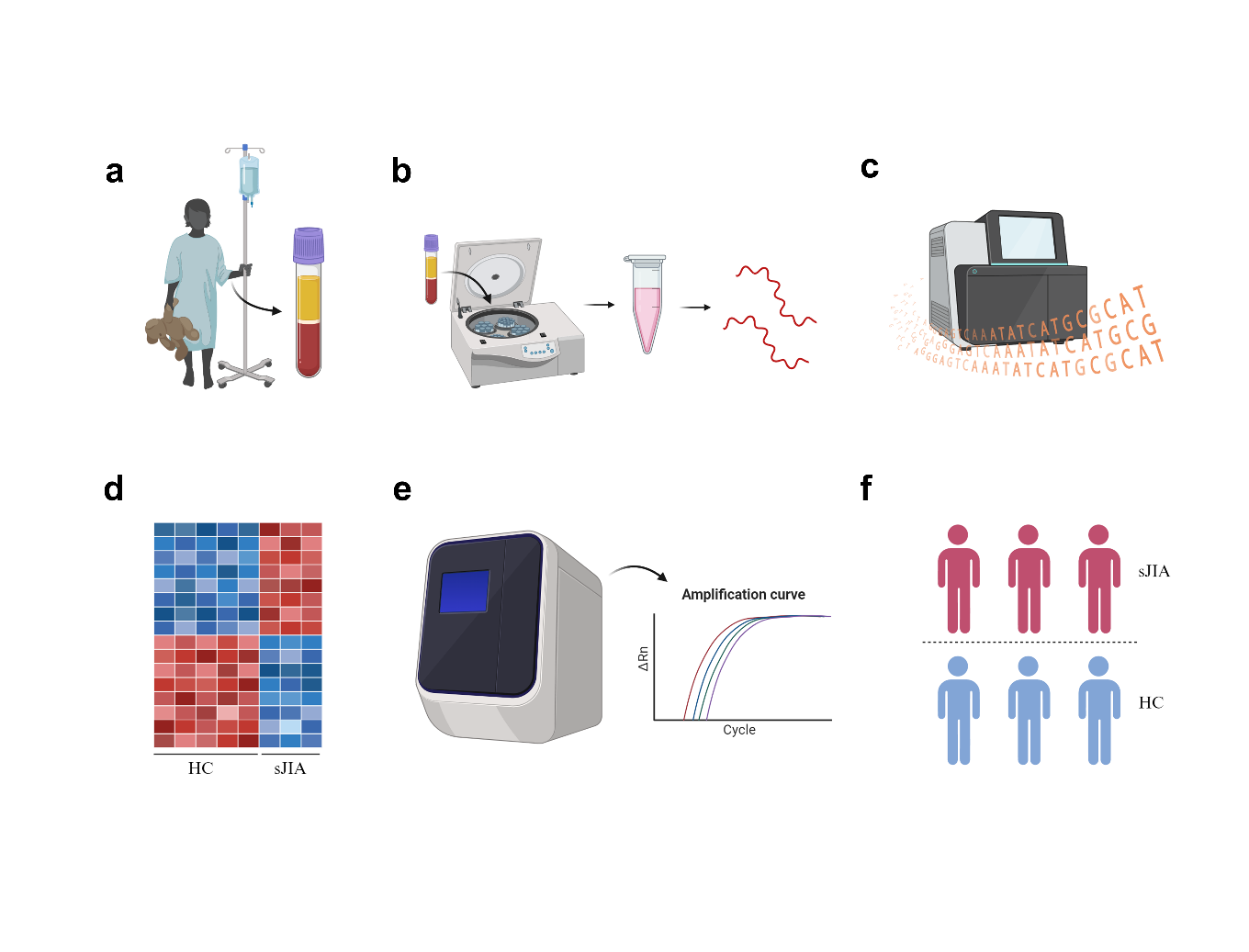


**Figure S1.** Schematic illustration to identify plasma tRFs biomarkers in sJIA. (a) Plasma samples were collected from sJIA patients. (b) RNA extraction from plasma samples using TRIzol. (c) Small-RNA sequencing. (d) Differential analysis for discovery of sJIA biomarker. (e) qRT-PCR validation of sJIA-related tRF dysregulation. (f) Fast screening and detection of sJIA based on selected tRF


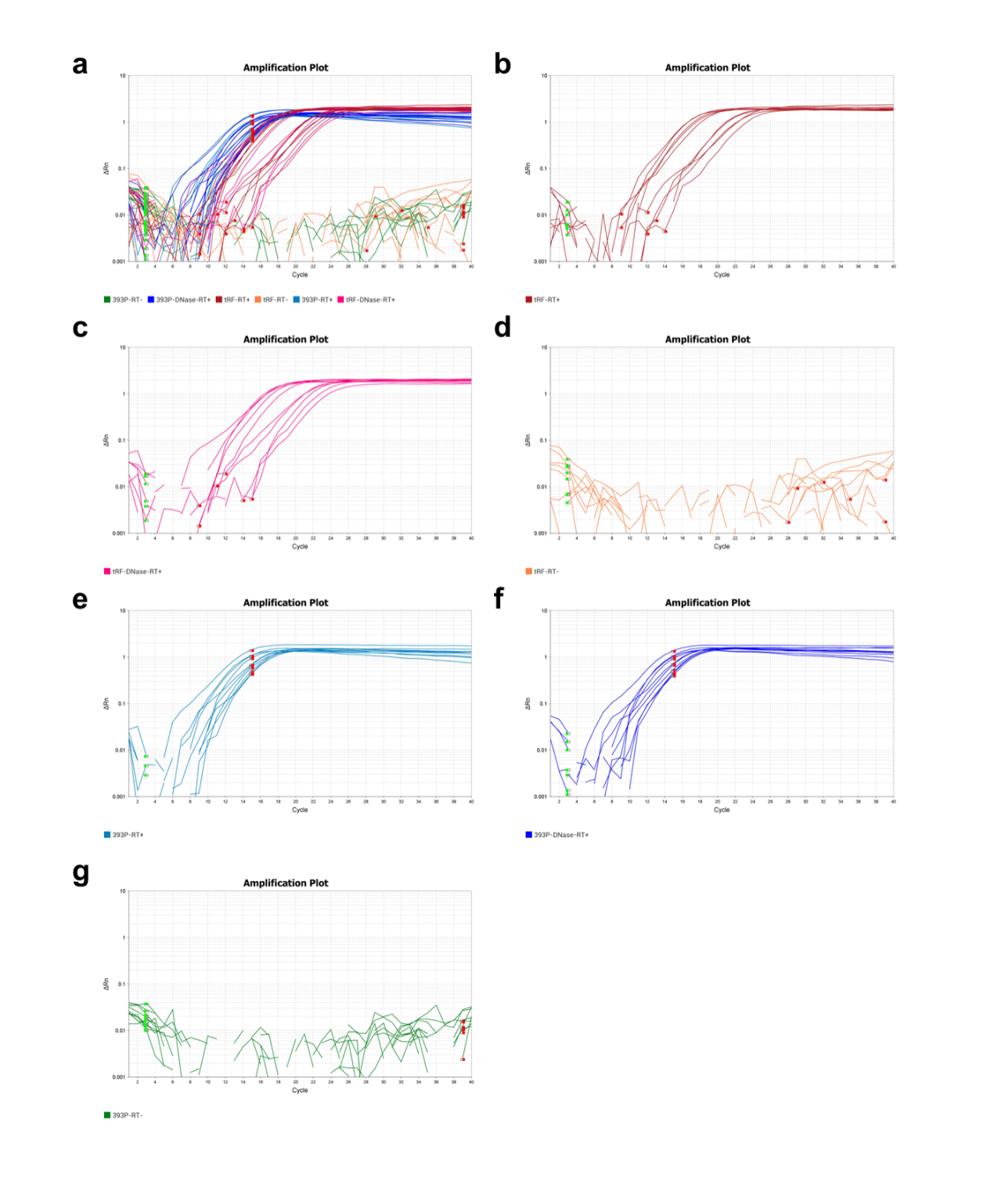
**Figure S2.** Representative amplification curves for DNase-treated and untreated RT+ reactions compared with RT− controls. (a) Overlay of all amplification curves for both target genes across all experimental conditions. (b–d) Amplification curves of tRF-30-FP18LPMBQ4NK for three conditions: +RT (no DNase) (b), +RT (DNase-treated) (c), and −RT (no DNase) (d). (e–g) Amplification curves of cel-miR-39-3p for the same three conditions: +RT (no DNase) (e), +RT (DNase-treated) (f), and −RT (no DNase) (g).


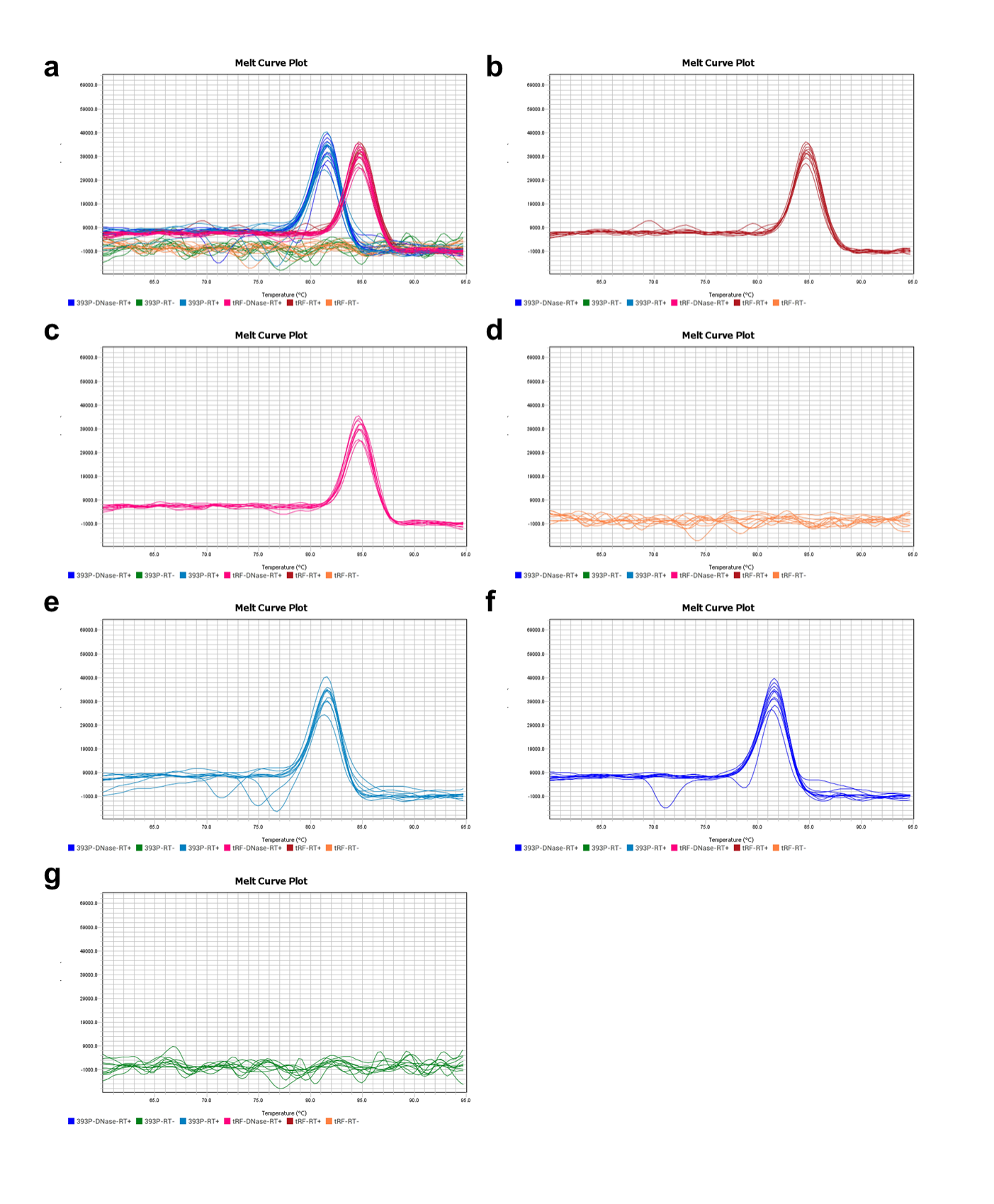
**Figure S3.** Representative melt curves for DNase-treated and untreated RT+ reactions compared with RT− controls. (a) Overlay of all melt curves for both target genes across all experimental conditions. (b–d) Melt curves of tRF-30-FP18LPMBQ4NK under three conditions: +RT (DNase-treated) (b), +RT (no DNase) (c), and −RT (no DNase) (d). (e–g) Melt curves of cel-miR-39-3p under the same three conditions: +RT (DNase-treated) (e), +RT (no DNase) (f), and −RT (no DNase) (g).

Table S1. The characters of patients with sJIA and HCs

|  | Training cohort | | | Validation cohort | | |
| --- | --- | --- | --- | --- | --- | --- |
| Index | sJIA(n=79) | HCs(n=52) | *p*-value | sJIA(n=30) | HCs(n=20) | *p*-value |
| Male/Female | 39/40 | 24/28 | 0.7187 | 14/16 | 9/11 | 0.9078 |
| Age | 9.48±3.41 | 9.56±3.30 | 0.8909 | 9.26±2.93 | 8.84±2.91 | 0.6199 |

Table S2. The clinical characters of patients with sJIA

|  | Training cohort | | | Validation cohort | | |
| --- | --- | --- | --- | --- | --- | --- |
| Index | Group A(n=27) | Group B(n=52) | *p*-value | Group C(n=10) | Group D(n=20) | *p*-value |
| Male/Female | 14/13 | 25/27 | 0.7503 | 4/6 | 10/10 | 0.6048 |
| Age | 9.38±3.20 | 9.53±3.54 | 0.8554 | 8.96±2.89 | 9.41±3.01 | 0.6986 |
| CRP(g/L) | 65.78±38.96 | 0.90±1.40 | <0.0001**** | 72.34±38.46 | 1.03±3.03 | <0.0001**** |
| WBC(*10^9^/L) | 16.30±6.21 | 7.82±2.20 | <0.0001**** | 16.60±4.62 | 6.87±1.64 | <0.0001**** |
| NE(*10^9^/L) | 12.98±5.90 | 4.32±1.86 | <0.0001**** | 13.08±4.18 | 3.23±0.74 | <0.0001**** |
| PLT(*10^9^/L) | 367.14±97.42 | 310.19±77.76 | 0.0062** | 335.90±82.33 | 306.55±47.73 | 0.2247 |
| ESR (mm/h) | 55.95±24.80 | 6.52±4.05 | <0.0001**** | 61.90±28.12 | 4.65±3.18 | <0.0001**** |
| SF(ng/mL) | 1117.844±1632.22 | 40.12±29.89 | <0.0001**** | 1175.56±440.53 | 41.73±21.97 | <0.0001**** |
| ALT(U/L) | 36.29±65.65 | 17.02±9.30 | 0.0422* | 23.10±13.65 | 16.00±6.21 | 0.0580 |
| TG(mmol/L) | 1.24±0.62 | 0.94±0.38 | 0.0428* | 1.39±0.46 | 0.89±0.44 | 0.0068** |
| Fib(g/L) | 4.49±0.75 | 2.69±1.05 | <0.0001**** | 4.70±0.33 | 2.85±0.87 | <0.0001**** |
| RF(IU/L) | ＜9.13 | ＜9.13 | / | ＜9.13 | ＜9.13 | / |
| Anti-CCP(IU/L) | ＜7 | ＜7 | / | ＜7 | ＜7 | / |
| IL-6(pg/mL) | 52.89±84.99 | / | / | 182.48±281.07 |  |  |
| IL-1β(pg/mL) | 7.79±6.87 | / | / | 4.774±3.57 |  |  |
| IL-10(pg/mL) | 2.52±1.63 | / | / | 10.11±5.60 |  |  |
| IFN-γ(pg/mL) | 12.97±7.18 | / | / | 7.60±7.97 |  |  |
| TNF-α(pg/mL) | 5.19±4.74 | / | / | 3.47±0.98 |  |  |
| IgG (g/L) | 11.34±3.76 | / | / | 11.36±3.09 |  |  |
| IgA (g/L) | 1.74±0.72 | / | / | 2.66±1.25 |  |  |
| IgM (g/L) | 1.40±0.54 | / | / | 1.36±0.54 |  |  |
| C3 (g/L) | 1.41±0.32 | / | / | 1.42±0.38 |  |  |
| C4 (g/L) | 0.31±0.11 | / | / | 0.33±0.09 |  |  |
| sJADAS27 | 12.56±6.55 | 0 | <0.0001**** | 13.29±4.25 | 0 | <0.0001**** |

CRP, C-reactive protein; WBC, white blood cells; NE, neutrophilic granulocyte; PLT, blood platelet; ESR, erythrocyte sedimentation rate; SF, serum ferritin; ALT, alanine aminotransferase; TG, triglycerides; Fib, fibrinogen; RF, rheumatoid factor; Anti-CCP, anti-cyclic citrullinated peptide; IgG, immunoglobulin G; IgA, immunoglobulin A, IgM, immunoglobulin M, C3, complement 3; C4, Complement 4; sJADAS27, systemic-onset Juvenile Arthritis Disease Activity Score 27-joint reduced count.

Table S3. The sequence of selected tRFs in patients with sJIA.

| tRF-ID | Sequence | Type | Length, bp | Regulation | Parental tRNA sequence |
| --- | --- | --- | --- | --- | --- |
| tRF-30-FP18LPMBQ4NK | AGCAGAGTGGCGCAGCGGAAGCGTGCTGGG | tRF-5c | 30 | UP | AGCAGAGTGGCGCAGCGGAAGCGTGCTGGGCCCATAACCCAGAGGTCGATGGATCGAAACCATCCTCTGCTACCA |
| tRF-30-M2OSRNLNKSEK | CGGGAGACCGGGGTTCGATTCCCCGACGGG | Other | 30 | UP | TCCTCGTTAGTATAGTGGTGAGTATCCCCGCCTGTCACGCGGGAGACCGGGGTTCGATTCCCCGACGGGGAGCCA |
| tRF-23-897PVP94Z | TCCTCGTTAGTATAGTGGTGAGT | tRF-5b | 23 | DOWN | TCCTCGTTAGTATAGTGGTGAGTATCCCCGCCTGTCACGCGGGAGACCGGGGTTCGATTCCCCGACGGGGAGCCA |
| tRF-28-79MP9P9NH50E | GTTTCCGTAGTGTAGTGGTTATCACGTT | tRF-5c | 28 | DOWN | GTTTCCGTAGTGTAGTGGTTATCACGTTCGCCTAACACGCGAAAGGTCCCCGGTTCGAAACCGGGCGGAAACACCA |

Table S4. Ct values for RT+ and RT− controls with and without DNase treatment.

| Sample ID | Group | Target | +RT (DNase) Ct | +RT (no DNase) Ct | -RT (no DNase) Ct | ΔCt (Dnase/no DNase) | ΔCt (-RT/+RT) |
| --- | --- | --- | --- | --- | --- | --- | --- |
| S1 | sJIA | tRF-30-FP18LPMBQ4NK | 14.303 | 14.256 | 39.274 | 0.047 | 25.017 |
| S2 | sJIA | tRF-30-FP18LPMBQ4NK | 13.347 | 12.851 | Undetermined | 0.496 | ＞22.149 |
| S3 | sJIA | tRF-30-FP18LPMBQ4NK | 13.679 | 13.389 | Undetermined | 0.289 | ＞21.611 |
| S4 | sJIA | tRF-30-FP18LPMBQ4NK | 14.146 | 13.480 | Undetermined | 0.665 | ＞21.520 |
| S5 | sJIA | tRF-30-FP18LPMBQ4NK | 14.873 | 15.278 | 35.165 | -0.406 | 19.886 |
| H1 | HC | tRF-30-FP18LPMBQ4NK | 18.620 | 18.702 | Undetermined | -0.082 | ＞16.298 |
| H2 | HC | tRF-30-FP18LPMBQ4NK | 18.602 | 19.375 | Undetermined | -0.773 | ＞15.625 |
| H3 | HC | tRF-30-FP18LPMBQ4NK | 17.918 | 17.669 | Undetermined | 0.249 | ＞17.331 |
| H4 | HC | tRF-30-FP18LPMBQ4NK | 19.867 | 20.155 | Undetermined | -0.288 | ＞14.845 |
| H5 | HC | tRF-30-FP18LPMBQ4NK | 17.782 | 17.046 | Undetermined | 0.736 | ＞17.954 |
| S1 | sJIA | cel-miR-39-3p | 12.680 | 12.397 | Undetermined | 0.283 | ＞22.603 |
| S2 | sJIA | cel-miR-39-3p | 13.324 | 12.798 | Undetermined | 0.526 | ＞22.202 |
| S3 | sJIA | cel-miR-39-3p | 11.729 | 11.031 | Undetermined | 0.699 | ＞23.969 |
| S4 | sJIA | cel-miR-39-3p | 13.199 | 13.081 | Undetermined | 0.118 | ＞21.919 |
| S5 | sJIA | cel-miR-39-3p | 13.343 | 12.786 | Undetermined | 0.557 | ＞22.214 |
| H1 | HC | cel-miR-39-3p | 10.971 | 10.424 | Undetermined | 0.547 | ＞24.576 |
| H2 | HC | cel-miR-39-3p | 9.640 | 8.894 | Undetermined | 0.746 | ＞26.106 |
| H3 | HC | cel-miR-39-3p | 11.485 | 11.289 | Undetermined | 0.197 | ＞23.711 |
| H4 | HC | cel-miR-39-3p | 11.111 | 10.248 | Undetermined | 0.864 | ＞24.752 |
| H5 | HC | cel-miR-39-3p | 12.241 | 11.817 | Undetermined | 0.424 | ＞23.183 |
